# Ischemia Enhances the Acute Stretch-Induced Increase in Calcium Spark Rate in Ventricular Myocytes

**DOI:** 10.3389/fphys.2020.00289

**Published:** 2020-04-16

**Authors:** Breanne A. Cameron, Hiroaki Kai, Keiko Kaihara, Gentaro Iribe, T. Alexander Quinn

**Affiliations:** ^1^Department of Physiology and Biophysics, Dalhousie University, Halifax, NS, Canada; ^2^Graduate School of Medicine, Dentistry, and Pharmaceutical Sciences, Okayama University, Okayama, Japan; ^3^Department of Physiology, Asahikawa Medical University, Asahikawa, Japan; ^4^School of Biomedical Engineering, Dalhousie University, Halifax, NS, Canada

**Keywords:** calcium, ischemia, myocytes, reactive oxygen species, stretch, ventricle

## Abstract

**Introduction:** In ventricular myocytes, spontaneous release of calcium (Ca^2+^) from the sarcoplasmic reticulum via ryanodine receptors (“Ca^2+^ sparks”) is acutely increased by stretch, due to a stretch-induced increase of reactive oxygen species (ROS). In acute regional ischemia there is stretch of ischemic tissue, along with an increase in Ca^2+^ spark rate and ROS production, each of which has been implicated in arrhythmogenesis. Yet, whether there is an impact of ischemia on the stretch-induced increase in Ca^2+^ sparks and ROS has not been investigated. We hypothesized that ischemia would enhance the increase of Ca^2+^ sparks and ROS that occurs with stretch.

**Methods:** Isolated ventricular myocytes from mice (male, C57BL/6J) were loaded with fluorescent dye to detect Ca^2+^ sparks (4.6 μM Fluo-4, 10 min) or ROS (1 μM DCF, 20 min), exposed to normal Tyrode (NT) or simulated ischemia (SI) solution (hyperkalemia [15 mM potassium], acidosis [6.5 pH], and metabolic inhibition [1 mM sodium cyanide, 20 mM 2-deoxyglucose]), and subjected to sustained stretch by the carbon fiber technique (~10% increase in sarcomere length, 15 s). Ca^2+^ spark rate and rate of ROS production were measured by confocal microscopy.

**Results:** Baseline Ca^2+^ spark rate was greater in SI (2.54 ± 0.11 sparks·s^−1^·100 μm^−2^; *n* = 103 cells, *N* = 10 mice) than NT (0.29 ± 0.05 sparks·s^−1^·100 μm^−2^; *n* = 33 cells, *N* = 9 mice; *p* < 0.0001). Stretch resulted in an acute increase in Ca^2+^ spark rate in both SI (3.03 ± 0.13 sparks·s^−1^·100 μm^−2^; *p* < 0.0001) and NT (0.49 ± 0.07 sparks·s^−1^·100 μm^−2^; *p* < 0.0001), with the increase in SI being greater than NT (+0.49 ± 0.04 vs. +0.20 ± 0.04 sparks·s^−1^·100 μm^−2^; *p* < 0.0001). Baseline rate of ROS production was also greater in SI (1.01 ± 0.01 normalized slope; *n* = 11, *N* = 8 mice) than NT (0.98 ± 0.01 normalized slope; *n* = 12, *N* = 4 mice; *p* < 0.05), but there was an acute increase with stretch only in SI (+12.5 ± 2.6%; *p* < 0.001).

**Conclusion:** Ischemia enhances the stretch-induced increase of Ca^2+^ sparks in ventricular myocytes, with an associated enhancement of stretch-induced ROS production. This effect may be important for premature excitation and/or in the development of an arrhythmogenic substrate in acute regional ischemia.

## Introduction

In cardiac myocytes, calcium (Ca^2+^) sparks represent the elementary release of Ca^2+^ from the sarcoplasmic reticulum (SR, the cell's primary Ca^2+^ store) via ryanodine receptors (RyR), and thus play a critical role in intracellular Ca^2+^ handling. The frequency of Ca^2+^ sparks is determined by RyR open probability, which under normal conditions is primarily influenced by the free intracellular Ca^2+^ concentration ([Ca^2+^]_i_) and the concentration of Ca^2+^ in the SR (Cheng and Lederer, 2008). In pathological settings, an elevation in Ca^2+^ spark rate, due to increased [Ca^2+^]_i_ or altered RyR function [secondary to metabolic, adrenergic, or genetic changes, or to channel phosphorylation, oxidation, or nitrosylation (Prosser et al., 2010)], has been implicated in the induction of aberrant Ca^2+^ waves and deadly cardiac arrhythmias (Ter Keurs and Boyden, 2007).

Recently, it has been shown that stretch of single ventricular myocytes can also acutely increase Ca^2+^ spark rate (Iribe et al., 2009), which has been suggested to perhaps “tune” excitation-contraction coupling in the whole heart (Prosser et al., 2013a), thus contributing to the Frank Starling mechanism (Quinn and Kohl, 2016) and acting to maintain contractile homogeneity across the ventricles by normalizing inter-cellular contractile force (Cannell, 2009; Quinn, 2015). Follow up studies have shown that the acute stretch-induced increase in Ca^2+^ sparks results from a microtubule-dependent increase in reactive oxygen species (ROS) production by NADPH oxidase 2 (NOX2) with stretch, which has been termed X-ROS (Prosser et al., 2011). Further, Ca^2+^ spark rate and X-ROS production have been shown to be enhanced by cyclic stretch (as occurs during filling with each heartbeat) and graded by stretch amplitude and frequency, which may additionally tune the mechanical activity and redox state of cardiac myocytes to changes in physiological demand (Prosser et al., 2013b).

An acute stretch-induced increase in Ca^2+^ sparks may be particularly relevant in disease states associated with heterogeneous changes in the heart's mechanical activity (Quinn, 2014). In patients suffering from acute regional ischemia, there is stretch of ischemic tissue, with the magnitude of stretch relating to the prevalence of ventricular fibrillation (Hirche et al., 1987; Barrabes et al., 1998, 2002, 2013, 2017). Moreover, there is a correlation between wall motion abnormalities and arrhythmia incidence in ischemia (Califf et al., 1978; Siogas et al., 1998), with ectopic excitation often originating from regions where systolic segment lengthening occurs (Lab, 1989; Coronel et al., 2002). In experimental models of non-uniform contraction, on the other hand, there is an increase in ROS production localized to stretched regions, which results in the activation of Ca^2+^ waves (Miura et al., 2018). Thus, in ischemia, a stretch-induced increase in Ca^2+^ sparks may act as a trigger, or contribute to the substrate, for the associated arrhythmias (Janse and Wit, 1989).

Myocardial ischemia is also associated with an increase in [Ca^2+^]_i_ (Baumeister and Quinn, 2016), leading to a rise in Ca^2+^-calmodulin-dependent protein kinase II (CaMKII) activity (Mattiazzi et al., 2015b), which increases phosphorylation of RyR and the frequency of Ca^2+^ sparks (Maier and Bers, 2007). Computational modeling has suggested that an elevation in [Ca^2+^]_i_ associated with hyperkalemia-induced membrane depolarization, along with a consequential reduction in NCX-mediated Ca^2+^ efflux and an increase in SR Ca^2+^ content may also contribute to an increase in Ca^2+^ spark rate in ischemia (Sato et al., 2014). Ca^2+^ spark rate is further increased in ischemia by an increase in ROS (Raedschelders et al., 2012; Köhler et al., 2014), including a contribution of increased NOX2 activity (Donoso et al., 2014), which increases the open probability of RyR by *S*-glutathionylation (Sánchez et al., 2005), and sensitizes RyR to cytoplasmic Ca^2+^ (Belevych et al., 2009; Zhang et al., 2013). In fact, an increased Ca^2+^ spark rate has been shown to be a contributor to the elevation in [Ca^2+^]_i_ in ischemia (Mattiazzi et al., 2015a). At the same time, ischemia is associated with a reduction of the key anti-oxidant glutathione (Ferrari et al., 1985; Poluektov et al., 2019), which computational modeling has suggested may increase the effects of stretch-induced ROS on Ca^2+^ sparks by limiting cellular reducing capacity through a decrease in glutathione, resulting in a consequential increase in the open probability of RyR during stretch (Limbu et al., 2015). This combination of effects on RyR activity and its modulators suggests that ischemia may alter the response of Ca^2+^ sparks to stretch, yet the effect of ischemia on the stretch-induced increase in Ca^2+^ spark rate has not been investigated.

We hypothesized that in simulated ischemic conditions, there would be an enhancement of the stretch-induced increase in Ca^2+^ sparks, with an associated potentiation of the stretch-induced increase in ROS production. Single ventricular myocytes isolated from mice were subjected to a single controlled stretch using carbon fibers in normal or simulated ischemic conditions (Iribe et al., 2009). Fluorescent confocal microscopy was used to monitor [Ca^2+^]_i_ or ROS, to assess the effect of stretch on Ca^2+^ spark rate and ROS production. Our results demonstrate that ischemia enhances the stretch-induced increase of Ca^2+^ spark rate in isolated ventricular myocytes, with an associated enhancement of stretch-induced ROS production. This enhancement of the stretch-induced increase in Ca^2+^ sparks may be a contributing factor to the lethal arrhythmias that occur in the setting of ischemia.

## Materials and Methods

Experiments were performed in accordance with the Guidance Principles for the Care and Use of Animals established by the Council of the Physiological Society of Japan. The experimental protocol was reviewed and accepted by the Animal Subjects Committee of Okayama University Graduate School of Medicine, Dentistry, and Pharmaceutical Sciences. Details of experimental protocols have been reported following the Minimum Information about a Cardiac Electrophysiology Experiment (MICEE) reporting standard (Quinn et al., 2011).

### Ventricular Myocyte Isolation

Ventricular myocytes were isolated from mice as previously described (Iribe et al., 2013, 2014). Mice (male C57BL/6J, 8–12 weeks old) were administered an intraperitoneal injection of heparin sodium (100 I.U.; Wockhardt, Mumbai, India). After 30 min, surgical anesthesia was induced by inhalation of isoflurane (IsoFlu; Abbott Laboratories, Abbott Park, USA) followed by rapid cardiac excision, aortic cannulation, and Langendorff perfusion for enzymatic cell isolation. Hearts were perfused at a rate of 4 mL/min for 3 min with Ca^2+^-free solution (composition in mM: 128 NaCl, 2.6 KCl, 1.18 MgSO_4_, 1.18 KH_2_PO_4_, 10 HEPES, 20 Taurine, 11 Glucose; pH 7.47 adjusted with NaOH), followed by 5-6 min of perfusion with 30 mL of Ca^2+^-free solution that included 6 mg of the enzyme blend Liberase TM Research Grade (Roche, Basel, Switzerland). The ventricles were harvested by cutting along the atrioventricular border, and then cut into 1–2 mm^3^ cubes and gently agitated in oxygenated Ca^2+^-free solution. The supernatant containing ventricular cells was collected, the remaining tissue was resuspended in fresh Ca^2+^-free solution, and the above procedure was repeated in triplicate. The collected supernatant was passed through a nylon mesh and centrifuged at 15 × g for 3 min, followed by resuspension of the resulting cell pellet in normal Tyrode's solution (NT, composition in mM: 140 NaCl, 5.4 KCl, 1.8 CaCl_2_, 1 MgCl_2_, 5 HEPES, 11 Glucose; pH 7.4 adjusted with NaOH). Cells were kept at room temperature (~22°C) until ready to be used.

### Single Cell Stretch

Two groups of quiescent ventricular myocytes were considered: a control group (exposed to NT) and a simulated ischemia (SI) group [exposed to an ischemic solution containing [in mM]: 140 NaCl, 15 KCl, 1.8 CaCl_2_, 1 MgCl_2_, 10 HEPES, 1 NaCN, 20 2-deoxyglucose, and with pH adjusted to 6.5 with NaOH (Murata et al., 2001; Khokhlova et al., 2017)], which mimicked the ~30 min time point of ischemia (phase 1b) through hyperkalemia [15 mM extracellular potassium], extracellular acidosis [pH 6.5], and metabolic inhibition (block of oxidative phosphorylation with 1 mM NaCN and inhibition of anaerobic glycolysis with 20 mM 2-deoxyglucose). Within a 30 min window from the beginning of exposure to SI solution, healthy cells (rod-shaped with clear striations, an intact membrane with no signs of blebbing, and no spontaneous intracellular Ca^2+^ waves before stretch) were subjected to a controlled, axial stretch at a single time point using the carbon fiber technique, as has been previously described for axial stretch of single ventricular myocytes (Iribe et al., 2007, 2009). Cells were placed on coverslips coated with poly-2-hydroxyethyl methacrylate (poly-HEMA; Sigma-Aldrich, Tokyo, Japan) to prevent cellular adhesion. Carbon fibers (10 μm in diameter) mounted in glass capillaries that adhere to isolated cells through biophysical interactions (Peyronnet et al., 2017) were attached to either end of an individual cell. For experiments examining the effect of stretch on Ca^2+^ sparks, one “compliant” (1.2 mm) and one “stiff” (0.6 mm) carbon fiber were used, while for the ROS experiments two compliant (1.2 mm) carbon fibers were used (Tsukuba Material Information Laboratory, Tsukuba, Japan). Tri-axial positioning of the carbon fibers was performed by custom-made three-axis hydraulic manipulators (Narishige, Tokyo, Japan), with the compliant carbon fibers mounted on piezo-electric translators (P-621.1 CL; Physik Instrumente, Karlsruhe, Germany) and the stiff carbon fiber held stationary. While the unidirectional stretch using one stiff stationary and one compliant translating carbon fiber was sufficient for the Ca^2+^ spark experiments, this produced too much motion of the cell under the detector during stretch for ROS production to be measured. The bi-directional stretch using two compliant translating carbon fibers reduced the level of cell motion, so allowed for more accurate measurements of ROS. Whole-cell axial stretch was applied by a 20 μm increase in the separation of the piezo-electric translators and held for 15 s, controlled by custom-written LabView software and a fast analog-to-digital converter (NI USB-6259; National Instruments, Austin, USA), which has been shown previously to result in a ~10% increase in sarcomere length (Iribe et al., 2017).

### Ca^2+^ Spark Measurement

Detection of Ca^2+^ sparks in ventricular myocytes was performed similar to a previously described technique (Iribe et al., 2009). Cells were incubated with Fluo-4-AM (4.6 μM, 10 min; Invitrogen, Carlsbad, California). Confocal images (XY, 30 fps, CSU-X1; Yokogawa, Tokyo, Japan) were obtained by excitation with a 488 nm laser and fluorescence emission detection above 505 nm. Ca^2+^ sparks were detected in the cellular region between the carbon fibers using custom LabVIEW software. Ca^2+^ spark rate was calculated by counting the number of sparks detected over 5 s, excluding duplicate counts by subtracting single-coordinate spark fluorescence that exceeded one contiguous frame, and was normalized by area (to control for variability in cell size) and reported as sparks·s^−1^·100 μm^−2^. The rate of image acquisition limited additional measurements of spark dynamics. Ca^2+^ spark rate was measured over three 5 s intervals: immediately before stretch, during stretch (immediately after its application), and immediately following complete release of stretch. Any cell in which an intracellular Ca^2+^ wave or synchronized SR Ca^2+^ release occurred during the analysis window was excluded (8% of cells in both conditions).

### ROS Measurement

The rate of intracellular ROS production was measured using 2′,7′-dichlorofluorescein diacetate (DCF) as previously described (Iribe et al., 2017). Cells were loaded with DCF (1 μm, 20 min; Life Technologies Japan, Tokyo, Japan) and confocal images (FV1000; Olympus Corporation, Tokyo, Japan) were captured (XY, 1.27 fps) by excitation with a 488 nm laser and fluorescence emission detection above 500 nm. As DCF fluorescence is known to be artificially amplified by continuous light exposure (Prosser et al., 2011, 2013b), a low laser intensity was used. The rate of ROS production was measured in the cellular region between the carbon fibers with custom LabView software as the slope of the increase in fluorescence over three 15 s intervals: immediately before stretch, during stretch, and immediately following complete release of stretch.

### Statistics

Data are presented as mean ± standard error of the mean (SEM). For the Ca^2+^ spark rate measurements, as the data was not normally distributed, Kruskal-Wallis test with *pot hoc* Dunn's multiple comparisons test was used to assess Ca^2+^ spark rate over time, and Wilcoxon paired or Mann-Whitney unpaired tests were used for comparison of group means. For the ROS production measurements, two-tailed paired or unpaired Student's *t*-tests were used for comparison of group means. A *p* < 0.05 was considered statistically significant.

## Results

### Effect of Stretch on Ca^2+^ Spark Rate

**Figure 1:**
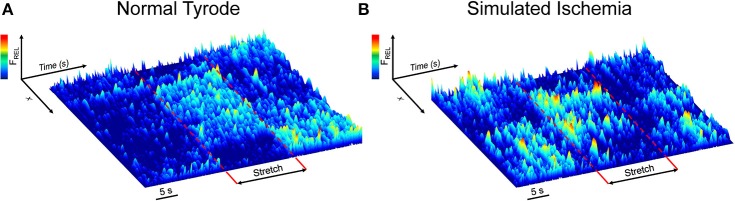
Effect of axial stretch on the occurrence of calcium sparks in single quiescent mouse ventricular myocytes exposed to either normal Tyrode **(A)** or simulated ischemia **(B)**. Fluorescence surface plots are derived from a line of pixels across the cell, providing a temporal depiction of the relative Fluo-4 fluorescence (F_REL_) before, during, and after 15 s of stretch (dashed red lines). Movies of the cells are provided as Supplemental Videos 1, 2.

Figure 1 shows surface plots of Fluo-4 fluorescence derived from a line of pixels across the confocal images of a cell, demonstrating the effect of stretch on the occurrence of Ca^2+^ sparks in single mouse ventricular myocytes exposed to either NT (Figure 1A) or SI (Figure 1B; movies of the fluorescence in the represented cells are presented in Supplemental Videos 1, 2). Ca^2+^ sparks occur more frequently in SI than NT and acutely increase in both groups during stretch, with a return to normal levels after stretch release.

**Figure 2:**
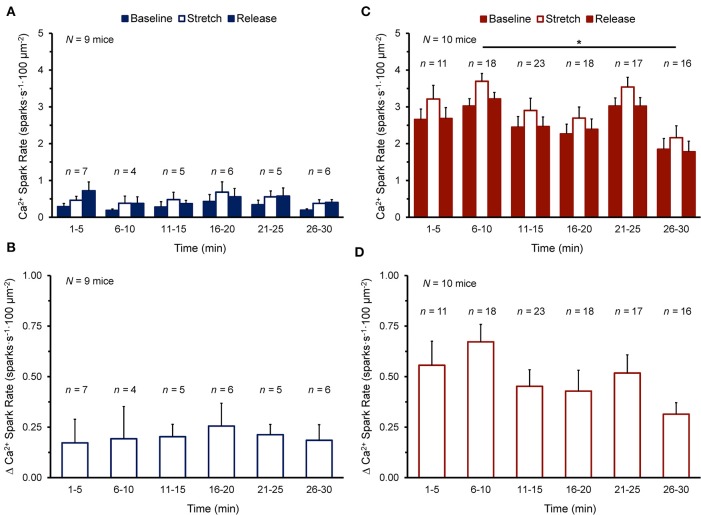
Effect of duration of exposure of quiescent mouse ventricular myocytes to normal Tyrode **(A,B)** or simulated ischemia solution **(C,D)** on calcium (Ca^2+^) spark rate before (Baseline), during (Stretch), and after stretch (Release) **(A,C)**, as well as the change in Ca^2+^ spark rate with stretch [Δ Ca^2+^ Spark Rate; **(B,D)**] over the 30 min measurement window. Cells were subjected to stretch at a single time point and values from all cells stretched within 5 min intervals were averaged (presented as mean ± SEM). *N* indicates the number of mice; *n* indicates the number of cells. *p < 0.01 by *post-hoc* Dunn's multiple comparisons test.

Figure 2 shows the Ca^2+^ spark rate before, during, and after stretch, and the change with stretch, in cells of the NT or SI group over the 30 min measurement window. Cells were subjected to stretch at a single time point and values from all cells stretched within 5 min intervals were averaged. There was no effect of time on Ca^2+^ spark rate (Figure 2A) or its change with stretch (Figure 2B) in the NT group, as values before, during, and after stretch and the change with stretch did not vary over the 30 min. There was variation of Ca^2+^ spark rate over 30 min in the SI group (Figure 2C; *p* < 0.05 by Kruskal-Wallis test), however values only differed between the 6-10 min and 26-30 min time points (*p* < 0.01, by *post-hoc* Dunn's multiple comparisons test), and there was no effect of time on the change in Ca^2+^ spark rate with stretch (Figure 2D). Thus, as the change in Ca^2+^ spark rate with stretch did not vary over the 30 min experimental window in either group, all cells in each group were averaged for further analysis.

**Figure 3:**
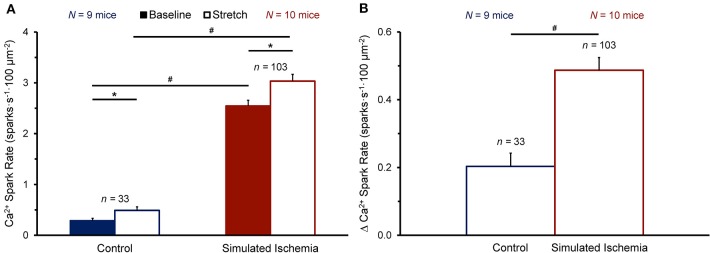
Effect of stretch of quiescent mouse ventricular myocytes on calcium (Ca^2+^) spark rate before (Baseline) and during stretch (Stretch; **A**) and the change in Ca^2+^ spark rate with stretch (Δ Ca^2+^ Spark Rate; **B**) averaged across all cells exposed to either normal Tyrode or simulated ischemia solution (presented as mean ± SEM). *p < 0.0001 by Wilcoxon paired test; ^#^*p* < 0.0001 by Mann-Whitney unpaired test. *N* indicates the number of mice; *n* indicates the number of cells.

Figure 3 shows the effect of stretch on Ca^2+^ spark rate averaged across all cells in the NT (*n* = 33 cells, *N* = 9 mice) or SI (*n* = 103 cells, *N* = 10 mice) group. The average Ca^2+^ spark rate was higher in SI than NT (Figure 3A), both at baseline (2.54 ± 0.11 vs. 0.29 ± 0.05 sparks·s^−1^·100 μm^−2^, *p* < 0.0001 by Mann-Whitney unpaired test) and during stretch (3.03 ± 0.13 vs. 0.49 ± 0.07 sparks·s^−1^·100 μm^−2^; *p* < 0.0001). With stretch, Ca^2+^ spark rate was acutely increased in both NT (+0.20 ± 0.04 sparks·s^−1^·100 μm^−2^; *p* < 0.0001 by Wilcoxon paired test) and SI (+0.49 ± 0.04 sparks·s^−1^·100 μm^−2^; *p* < 0.0001), with a larger increase in Ca^2+^ spark rate with stretch in SI compared to NT (*p* < 0.0001; Figure 3B).

### Effect of Stretch on ROS Production

**Figure 4:**
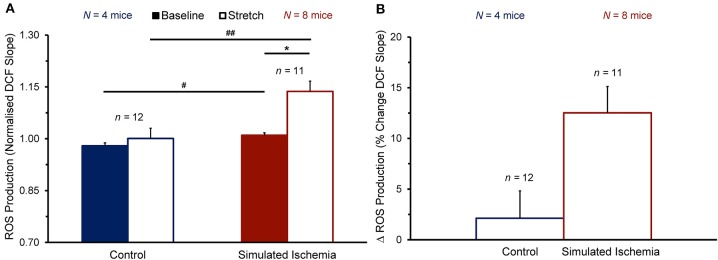
Effect of stretch of quiescent mouse ventricular myocytes on the rate of reactive oxygen species (ROS) production (quantified as the slope of the change in 2′,7′-dichlorofluorescein diacetate, DCF, fluorescence over time) before (Baseline) and during stretch (Stretch; **A**) and the change in ROS production with stretch (Δ ROS Production; **B**) averaged across all cells exposed to either normal Tyrode or simulated ischemia solution (presented as mean ± SEM). *p < 0.001 by two-tailed, paired Student's *t*-test; ^#^*p* < 0.05 and ^##^*p* < 0.01, by two-tailed, unpaired Student's *t*-test. *N* indicates the number of mice; *n* indicates the number of cells.

To determine whether the observed enhancement of the stretch-induced increase in Ca^2+^ sparks in SI compared to NT is associated with a concomitant enhancement of the stretch-induced increase in ROS production that has been shown by others (Prosser et al., 2011), cells were loaded with DCF to assess the rate of ROS production before and during stretch (measured as the slope of the change in DCF fluorescence over time). Figure 4 shows the effect of stretch on the rate of ROS production in cells exposed to NT (*n* = 12 cells, *N* = 4 mice) or SI (*n* = 11 cells, *N* = 8 mice). The rate of ROS production was greater in SI than in NT both at baseline (1.01 ± 0.01 vs. 0.98 ± 0.01 normalized slope; *p* < 0.05 by two-tailed unpaired Student's *t*-test) and during stretch (1.14 ± 0.03 vs. 1.00 ± 0.03; *p* < 0.01). With stretch, the rate of ROS production was acutely increased in SI (+12.5 ± 2.6%; *p* < 0.001 by two-tailed paired Student's *t*-test), but not in NT (+2.1 ± 2.7%).

## Discussion

This study sought to determine the effect of ischemia on the increase in Ca^2+^ spark rate and ROS production that occurs with stretch in ventricular myocytes. It was found that in simulated ischemic conditions, the basal level of both Ca^2+^ spark rate and ROS production were greater than in control, and that the increase in spark rate with stretch was enhanced in ischemic conditions. The difference in the response of Ca^2+^ spark rate to stretch represents a potential contributing mechanism to aberrant Ca^2+^ handling in ischemia and could be a source of premature excitation and the arrhythmogenic substrate in this pathological state.

### Mechanisms of Ventricular Arrhythmias in Acute Regional Ischemia

Acute regional ischemia is a major cause of sudden cardiac death (Zipes and Wellens, 1998; Rubart and Zipes, 2005), with lethal ventricular arrhythmias accounting for 80% of all cases without a prior history of heart disease (Myerburg et al., 1997). Arrhythmias in the first hour of ischemia [phase 1, during which ~50% of all sudden cardiac deaths occur (Di Diego and Antzelevitch, 2011)] are both focal and re-entrant in nature, resulting from a combination of ischemia-induced changes in electrical, mechanical, and biochemical properties of the myocardium (Janse and Wit, 1989; Carmeliet, 1999).

The arrhythmogenic changes in ventricular myocyte electrophysiology during acute ischemia have been extensively studied (Janse and Wit, 1989; Carmeliet, 1999). The most prominent effects include: (i) a decrease in ATP, pH, and the fast sodium and L-type Ca^2+^ currents; (ii) an increase in intracellular Ca^2+^ and sodium concentrations; and (iii) activation of the ATP-sensitive potassium current. At the same time, extracellular potassium concentration and catecholamine levels are also increased. All of the above changes (except for the increase in catecholamine levels), which have been shown to result in arrhythmogenic alterations of the ventricular action potential, including: (i) depolarization of the resting membrane potential; (ii) a decrease in the action potential upstroke, amplitude, and duration; and (iii) an increase in the effective refractory period, were simulated or present in the current study.

Other, poorly understood cell-level effects are thought to contribute to ventricular arrhythmias in acute ischemia, including cell stretch and changes in intracellular Ca^2+^ handling. The acute phase of ischemia is associated with tissue stretch, which may result in ectopic excitation (Lab, 1989; Coronel et al., 2002) and whose magnitude correlates with the prevalence of ventricular fibrillation (Hirche et al., 1987; Barrabes et al., 1998, 2002, 2013, 2017). At the same time, acute ischemia causes an increase in the frequency of Ca^2+^ sparks that contributes to an increase in [Ca^2+^]_i_ (Mattiazzi et al., 2015a). The increase in [Ca^2+^]_i_ in ischemia is thought to be arrhythmogenic (Baumeister and Quinn, 2016) by driving excitatory after-depolarizations (Billman et al., 1991; Xing and Martins, 2004; Wu et al., 2011). Thus, if links exist between cell stretch and altered Ca^2+^ handling in ischemia, they could represent important mechanisms for arrhythmogenesis.

### Effects of Stretch on Ca^2+^ Sparks

The frequency of Ca^2+^ sparks is determined by RyR open probability, which is strongly influenced by [Ca^2+^]_i_ and the concentration of Ca^2+^ in the SR (Cheng and Lederer, 2008). Previous studies have shown that in healthy conditions, stretch acutely increases the frequency of Ca^2+^ sparks in ventricular cells (Iribe et al., 2009), which is the result of an increase in NOX2-dependent ROS production (X-ROS), with microtubules acting as the mechano-transducers (Prosser et al., 2011). Interestingly, Ca^2+^ spark rate and X-ROS production are further increased by cyclic stretch (compared to sustained stretch), which is more similar to what occurs in the regularly beating heart (Prosser et al., 2013b). This response is also graded by the amplitude and frequency of stretch (Prosser et al., 2013b), so may help match the [Ca^2+^]_i_ and redox state of cardiac myocytes to changes in physiological demand.

In many diseases, there is an increase in the frequency of Ca^2+^ sparks, often due to an increase in [Ca^2+^]_i_ or altered RyR function [relating to cellular changes such as membrane depolarization (Sato et al., 2014) or increased ROS (Prosser et al., 2010)], which has the potential to lead to intracellular Ca^2+^ waves and cardiac arrhythmias (Ter Keurs and Boyden, 2007). This includes diseases in which there are changes in cardiac mechanics resulting in regions of tissue stretch (Quinn, 2014). For instance, in an experimental model of non-uniform myocardial contraction, it has been shown that there is an increase in ROS production localized to stretched regions, which can result in intracellular Ca^2+^ waves (Miura et al., 2018). Interestingly, in a similar experimental model, it has been shown that Ca^2+^ waves in fact relate to the rapid shortening of muscle immediately after a period of stretch (ter Keurs et al., 2006), due to a surge in intracellular Ca^2+^ as it dissociates from troponin-C (Wakayama et al., 2005). This Ca^2+^ surge is precipitated by a length-dependent increase in the affinity of troponin-C for Ca^2+^ (Allen and Kentish, 1988) and can result in after-depolarizations, premature excitation (Miura et al., 2008), and sustained arrhythmias (Miura et al., 2010).

In the present study, we observed an acute increase in basal Ca^2+^ spark rate in ischemia, which was further increased with stretch. This increase in Ca^2+^ spark rate with stretch was greater than that seen under normal conditions, representing an enhancement of the stretch-induced effect. Further, there was a higher baseline level of ROS production in ischemia compared to control, and an increase in ROS production with stretch in ischemia, which may in part account for the higher baseline levels of Ca^2+^ sparks and the increase with stretch in ischemia (although the difference in baseline level of ROS production between ischemia and control was small [3%], so may in fact not be physiologically significant).

While it is speculated that the observed enhancement of the stretch-induced increase in Ca^2+^ sparks in ischemic conditions may represent an arrhythmogenic mechanism, we observed few stretch-induced intracellular Ca^2+^ waves or synchronized SR Ca^2+^ releases, and the amount did not differ between ischemic and control cells (1 vs. 0% occurrence of intracellular Ca^2+^ waves; 7 vs. 5% occurrence of synchronized SR Ca^2+^ releases).

### Mechanisms of Enhanced Stretch Effects in Ischemic Conditions

The enhancement of the increase in Ca^2+^ sparks and ROS with stretch in ischemia may result from effects on various factors involved in the stretch-induced response (Ward et al., 2014; Joca et al., 2020). Ischemia may increase the mechano-sensitivity or responsiveness of NOX2, and mechano-transduction may be increased due to changes in microtubule properties, cell stiffness, or mechano-sensitive channel function (including transient receptor potential channels), resulting in greater X-ROS production with stretch. Alternatively (or additionally), rather than an increase in the production of X-ROS, its effect on Ca^2+^ sparks could be increased by a reduction of anti-oxidants in the cell [as suggested by computational modeling (Limbu et al., 2015)], as the key anti-oxidant glutathione is reduced in ischemia (Ferrari et al., 1985). At the same time, there could be a contribution of emergent, X-ROS-independent mechano-transduction pathways (e.g., via direct effects relating to the baseline increase in [Ca^2+^]_i_ or ROS with ischemia on mechano-sensitive ion channel activity, CaMKII activation, RyR sensitivity to Ca^2+^, or mitochondrial-derived Ca^2+^ and ROS production). These possibilities warrant further investigation, as they may represent novel anti-arrhythmic targets in ischemia.

### Comparison of Results to Previous Studies

In our study, under normal conditions we observed an increase in the Ca^2+^ spark rate with axial stretch of isolated mouse ventricular myocytes, as has been previously reported by others in both rat (Iribe et al., 2009; Prosser et al., 2011) and mouse (Prosser et al., 2011). We did not, however, see an associated increase in ROS production with stretch (~2%; measured as the change in the slope of DCF fluorescence), as shown in other studies (~70% for rat, ~40% for mouse) (Prosser et al., 2011). In ischemic conditions we did see a statistically significant stretch-induced increase in ROS production (~13%), along with a greater increase in Ca^2+^ spark rate than control (suggesting the increase in ROS might be involved), yet this increase was still much lower than the previously reported control values. The reason for this discrepancy is unclear. It may relate to methodological differences or differences in data handling. Our experiments involved the stretch of ventricular myocytes using 10 μm diameter compliant carbon fibers, which stick to the cells through biophysical interactions (Peyronnet et al., 2017). The previous work from Prosser et al. (2011) used 20 μm stiff glass rods adhered to the cells with a biological adhesive (Myotak, composed of laminin, entactin, heparin sulfate proteoglycan, gentamicin, Dulbecco's Modified Eagle Medium, collagen IV, Alexa Fluor-647 conjugated to bovine serum albumin, and an inert alumina silica aggregate with a diameter of 1 μm, dissolved in 100 μM BSA). While it was reported that none of these components are harmful to cells (as normal cell morphology and robust contractions are maintained for up to 2 h following cell attachment), it could be there are unappreciated effects of Myotak on ROS production, which could account for the greater effect in the previous work. Alternatively, differences in imaging methods (for instance whole cell confocal measurements in the current study vs. line scans in the previous report) or data analysis (such as measurement of DCF fluorescence slope by fitting a linear relation across the entire stretch period vs. the peak DCF slope extracted from a polynomial fit) might also be involved.

### Potential Limitations of the Current Study

In the current study, ischemia was simulated by a solution which mimicked the ~30 min time point of ischemia (phase 1b), including hyperkalemia, acidosis, and metabolic inhibition. Previous studies that exposed isolated ventricular myocytes to simulated ischemic conditions have used a similar approach but have simulated hypoxia by the use of a 90% nitrogen-10% carbon dioxide gas phase directed over the cell chamber (Cordeiro et al., 1994; O'Brien et al., 2008) or by bubbling the cell chamber with nitrogen (Tang et al., 2012) to displace oxygen from the solution. These techniques, however, are not compatible with the carbon fiber technique or our fluorescence imaging, as they cause mechanical disruption and motion artifact. As such, we simulated hypoxia with NaCN, which blocks oxidative phosphorylation, resulting in a lack of ATP generation, as reported previously by others (Murata et al., 2001; Khokhlova et al., 2017). One concern with this approach might be the effect of NaCN on cell viability. While we found that a majority of the cells exposed to our SI solution remained viable over the 30 min experimental period, to mitigate any concern that cells used were unhealthy, we included only myocytes that appeared rod-shaped with clear striations, had an intact membrane with no signs of blebbing, and displayed no spontaneous intracellular Ca^2+^ waves.

Temperature has been shown previously to affect Ca^2+^ sparks, with a higher spark rate, amplitude, and time to peak at room temperature compared to 37°C due to effects on sarcoplasmic reticulum Ca^2+^ stores and RyR function (Ferrier et al., 2003; Fu et al., 2005). While our experiments were performed at room temperature (~22°C), this is in-line with the previous studies of the effects of stretch on Ca^2+^ sparks (Iribe et al., 2009; Prosser et al., 2011), and it is not believed that that the associated difference in baseline spark rate is responsible for the fundamental effect of stretch on the frequency of Ca^2+^ sparks, nor the difference in the response of Ca^2+^ spark rate to stretch between NT and SI cells (although it may impact the magnitude of the response). Related to this concern, the increase in Ca^2+^ spark rate in ischemia compared to control, as well as its increase with stretch could be partly related to changes in intracellular Ca^2+^, however this was not measured in the current study (it would require the use of a ratiometric Ca^2+^- imaging approach).

## Conclusion

In the current study, we have demonstrated that ischemia enhances the acute increase in calcium spark rate that occurs with stretch of isolated ventricular myocytes, with an associated enhancement of stretch-induced ROS production. While the mechanism(s) of these enhancements remains unknown, they may contribute to the generation of Ca^2+^-induced arrhythmias in acute ischemia, and thus represent potential therapeutic targets for lethal arrhythmias.

## Data Availability Statement

The datasets generated for this study are available on request to the corresponding author.

## Ethics Statement

This animal study was reviewed and approved by Animal Subjects Committee of Okayama University Graduate School of Medicine, Dentistry, and Pharmaceutical Sciences.

## Author Contributions

BC contributed to the design of the study, performed the experimental work, analyzed the data, and wrote the manuscript. HK and KK provided technical support for the experiments. GI and TAQ contributed to the design of the study and revised the manuscript. All authors approved the final submission.

### Conflict of Interest

The authors declare that the research was conducted in the absence of any commercial or financial relationships that could be construed as a potential conflict of interest. The handling editor is currently organizing a Research Topic with one of the authors (GI) and confirms the absence of any other collaboration.

## Supplementary Material

Supplemental Video 1 Movie showing the effect of axial stretch on the occurrence of calcium sparks in single quiescent mouse ventricular myocytes exposed to normal Tyrode, measured by two-dimensional confocal microscopic recordings of Fluo-4AM fluorescence.

Supplemental Video 2 Movie showing the effect of axial stretch on the occurrence of calcium sparks in single quiescent mouse ventricular myocytes exposed to simulated ischemia, measured by two-dimensional confocal microscopic recordings of Fluo-4AM fluorescence.

## Abbreviations

Ca^2+^: calcium
CaMKII: Ca^2+^-calmodulin-dependent protein kinase II
DCF: 2′,7′-dichlorofluorescein diacetate
[Ca^2+^]_i_: free intracellular calcium concentration
NOX2: NADPH oxidase 2
NT: normal Tyrode
ROS: reactive oxygen species
RyR: ryanodine receptors
SI: simulated ischemia
SEM: standard error of the mean
SR: sarcoplasmic reticulum
X-ROS: NOX2-dependent ROS.
